# Design and Characterization of Inulin Conjugate for Improved Intracellular and Targeted Delivery of Pyrazinoic Acid to Monocytes

**DOI:** 10.3390/pharmaceutics11050243

**Published:** 2019-05-22

**Authors:** Franklin Afinjuomo, Thomas G. Barclay, Ankit Parikh, Yunmei Song, Rosa Chung, Lixin Wang, Liang Liu, John D. Hayball, Nikolai Petrovsky, Sanjay Garg

**Affiliations:** 1 School of Pharmacy and Medical Sciences, University of South Australia, Adelaide, SA 5001, Australia; olumide.afinjuomo@mymail.unisa.edu.au (F.A.); tom.barclay@unisa.edu.au (T.G.B.); ankit.parikh@unisa.edu.au (A.P.); may.song@unisa.edu.au (Y.S.); rosa.chung@unisa.edu.au (R.C.); lixin.wang@mymail.unisa.edu.au (L.W.); liang.liu@unisa.edu.au (L.L.); john.hayball@unisa.edu.au (J.D.H.); 2 Vaxine Pty. Ltd., Adelaide, SA 5042, Australia; nikolai.petrovsky@flinders.edu.au; 3 Department of Endocrinology, Flinders University, Adelaide, SA 5042, Australia

**Keywords:** inulin, pyrazinoic acid, intracellular delivery, tuberculosis

## Abstract

The propensity of monocytes to migrate into sites of mycobacterium tuberculosis (TB) infection and then become infected themselves makes them potential targets for delivery of drugs intracellularly to the tubercle bacilli reservoir. Conventional TB drugs are less effective because of poor intracellular delivery to this bacterial sanctuary. This study highlights the potential of using semicrystalline delta inulin particles that are readily internalised by monocytes for a monocyte-based drug delivery system. Pyrazinoic acid was successfully attached covalently to the delta inulin particles via a labile linker. The formation of new conjugate and amide bond was confirmed using zeta potential, Proton Nuclear Magnetic Resonance (^1^HNMR) and Fourier transform infrared spectroscopy (FTIR). Scanning electron microscopy (SEM) confirmed that no significant change in size after conjugation which is an important parameter for monocyte targeting. Thermogravimetric analysis (TGA) and differential scanning calorimetry (DSC) were used to establish the change in thermal properties. The analysis of in-vitro release demonstrated pH-triggered drug cleavage off the delta inulin particles that followed a first-order kinetic process. The efficient targeting ability of the conjugate for RAW 264.7 monocytic cells was supported by cellular uptake studies. Overall, our finding confirmed that semicrystalline delta inulin particles (MPI) can be modified covalently with drugs and such conjugates allow intracellular drug delivery and uptake into monocytes, making this system potentially useful for the treatment of TB.

## 1. Introduction

*Mycobacterium tuberculosis* (*Mtb*) remains a deadly and contagious human pathogen responsible for tuberculosis which is now the leading cause of death from infectious diseases surpassing human immunodeficiency virus infection (HIV) and acquired immune deficiency syndrome (AIDS) according to reports from the Global Tuberculosis Report 2017 [1]. Tuberculosis (TB) affects around 2 billion people or 30% of the global population making it a significant public health problem [2]. Despite the progress achieved in global TB control, eradication and treatment has been significantly jeopardized by the HIV epidemic [3,4,5] and the emergence of multidrug-resistant TB (MDR) and extensively drug-resistant TB (XDR TB) [6,7]. Intracellular pathogens such as *Mycobacterium tuberculosis* cause chronic infection in human because the pathogen has adapted specialized mechanisms of evading and overturning immune recognition, phagosome-lysosome fusion, and destruction by the immune system of the host [8,9,10]. Also, the pathogen uses infected monocytes as cellular reservoirs [11] and vehicles to spread to other parts of the body [12].

Unfortunately, the Bacille Calmette–Guérin (BCG) vaccine only protects children against disseminated infection but not pulmonary TB and doesn’t provide any protection of adults [13]. Furthermore, current conventional TB treatments are limited in their ability to reach target sites and to fully control both replicating and non-replicating bacteria in infected macrophages making treatment of TB very difficult. Due to its survival strategies the TB pathogen is capable of escaping destruction by therapeutic drugs [13], which translates to treatment failure and extended treatment regimens. Adding pyrazinamide (PZA), which uniquely kills dormant tubercle bacilli, to the drug regimen allows reduction in the duration of TB treatment from 9–12 months to about 6 months. PZA is a prodrug converted intracellularly into pyrazinoic acid (POA) by nicotinamidase enzyme [14]. The use of POA ester derivatives to deliver POA intracellularly has been reported [15,16]. However, the instability and hydrolysis of the POA ester before reaching the target site was a major setback during in-vivo studies despite a promising results in-vitro. Consequently, designing a stable POA prodrug with serum stability and good intracellular targeting remains a major challenge.

The host innate immunity plays a key role in protection against *Mtb.* Monocytes are a highly mobile subclass of the circulating white blood cells that are recruited from the bloodstream to site of infection, injury, and inflammation in the body [17,18]. The recruitment of monocytes is crucial for host defences against invading pathogens, but they also contribute to the pathogenesis of inflammatory diseases [17]. This recruitment, migration, ability to penetrate sites of inflammation, tumours and also cross biological barriers, reaching deep hypoxic areas [19,20]; this makes them an important target that can be utilised to deliver drug cargos in the treatment of conditions such as tuberculosis [21,22], HIV [23,24], cancer [25,26], and inflammatory diseases [27]. Chronic infections like TB could be alleviated by the design of novel drug delivery systems that use the body’s own circulating monocytes to safely deliver drugs intracellularly to the tubercle bacilli reservoir and is an approach that is currently gaining more attention [28].

Delivery of therapeutics to monocytes and macrophages has often exploited particle-based delivery strategies. Modification of the particles surface chemistries, attaching ligands to particles and using different shape and size of particles are different approaches reported in the literature as a strategy in the targeting of drugs to monocytes [29]. A type of particle previously used as a vaccine adjuvant is constructed from inulin, a natural plant fructan found in chicory, dahlia, and Jerusalem artichoke [30]. Inulin consists of linear chains of fructosyl groups linked by β (2→1) glyosidic bonds that is terminated at the reducing end by an α-d-(2→1)-glucopyranoside ring [31]. Semicrystalline form of inulin (MPI) can be exploited for targeting and sustained drug release to monocytes because this insoluble form of inulin prevents rapid renal excretion. Previous work from our group has shown that this semicrystalline inulin is approximately 1–2 microns in diameter with regular and consistent spherulite-like discoid structure [32]. Due to its potent ability to activate complement, this modified inulin has demonstrated potential use for cancer treatment [33] and as a vaccine adjuvant [34,35,36,37]. MPI has excellent tropism towards monocytes, and an ability to bind and be internalized by monocytes with high efficiency [38].

**Figure 1:**
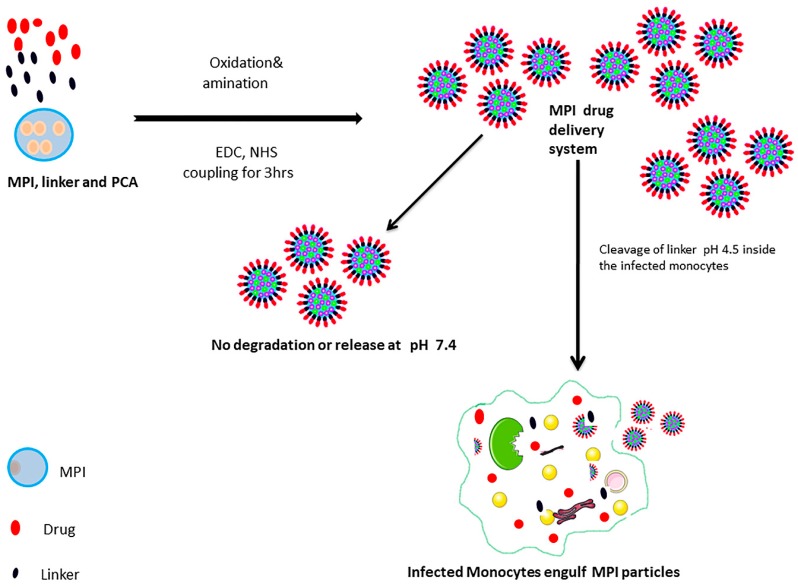
Schematic illustration of the MPI drug delivery system.

**Figure 2:**
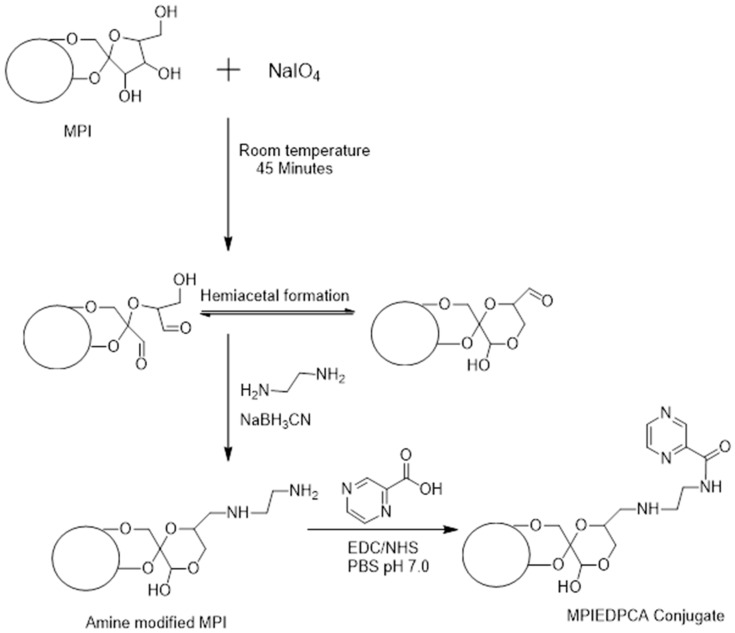
Synthesis of MPI conjugate.

This study involved the use of MPI as starting material for the development of a novel drug delivery system to target drugs to human immune cells. We hypothesized that the mononuclear phagocytes that act as the principal vehicle for dissemination of tubercle bacilli could also serve as a transporter for the delivery of TB drug. In order to improve the stability of the POA conjugates and cellular targeting of antibiotics to phagocytic cells, we reasoned that by attaching TB drugs to the inulin nanostructure surface by using a covalent hydrolysable linkage the conjugate will be cleaved by intracellular acidic conditions and enzymes contained in the lysosome after internalization into monocytes (Figure 1). To achieve this, we designed amine-modified MPI particles coupled to pyrazinoic acid (PCA) using carbodiimide reaction chemistry *N*-(3-dimethylaminopropyl)-*N*′-ethylcarbodiimide hydrochloride (EDC) and *N*-hydroxysuccinimide (NHS) that allows pH-triggered and controlled release of the active drug PCA into the cellular compartments including lysosomes (Figure 2). This should ultimately result in the release of drugs inside the infected monocytes.

This system exploits the change in pH that occur along the monocytes endosomal/lysosomal pathway from neutral extracellular pH of ~7.4 to an endosomal acidic pH of ~4.5. The modified MPI vehicle developed here allows the MPI to act as both a drug delivery system, and its carrier delta inulin itself has intrinsic immunostimulatory activity [39]. The safety profile, simple modification procedure, abundancy, biodegradability, and biocompatibility of inulin gives it several advantages over other targeting systems. The drug-modified MPI particles (MPIEDPCA) were characterized in this study using physicochemical techniques including FTIR, ^1^HNMR, as well as in-vitro drug release and cellular uptake study.

## 2. Materials and Methods

### 2.1. Materials

Delta inulin was prepared and supplied by our industrial partner Vaxine Pty Ltd. (Adelaide, Australia) by a method previously described [32]. PCA, sodium metaperiodate, sodium acetate, acetic acid, glycerol, ethanolamine, ethylenediamine, acetonitrile, sodium cyanoborohydride solution (5 M in 1 M NaOH), *N*-(3-dimethylaminopropyl)-*N*′-ethylcarbodiimide hydrochloride (EDC) and *N*-hydroxysuccinimide (NHS) and phosphate buffered saline tablets (PBS, pH 7.4, 0.01 M), 6-[Fluorescein-5(6) carboxamido] hexanoic acid (FITC) were purchased from Sigma-Aldrich Castle Hill, New South Wales Australia. Dimethyl sulfoxide (DMSO) for cell work were supplied by Merck (New South Wales, Australia). The NMR solvents, deuterated water (D_2_O) and DMSO (DMSO-D6), were purchased from Cambridge Isotope Laboratories (Tewksbury, MA, USA). All reagents and chemicals used were of analytic grade. General cell culture was supplied as a gift from Vaxine Pty Ltd. For the biological work, cell media and staining reagents Dulbecco’s Modified Eagle Medium (DMEM), l-Glutamine, trypsin and McCoy’s 5A (Modified) Medium were all purchased from Thermo Fisher Scientific (Thebarton, Adelaide, Australia). High purity (Milli-Q) water obtained from Sartorius™ Arium Pro-Ultrapure Water System was used in all of the experiments.

### 2.2. Experimental/Methods

#### Oxidation and Diamine Coupling of Inulin Particles

For inulin particle oxidation, sodium periodate (40 mg) was dissolved in sodium acetate buffer (0.1 M, pH 5.5) in 4 mL to give a concentration of 10 mg/mL in a light protected container. In a separate light protected container, inulin particles (3.4 mL, 53 mg/mL, 180 mg) were added to 10.7 mL sodium acetate buffer to make up 14.1 mL in a light protected container. The sodium periodate solution (3.92 mL) was added to the inulin. The final concentration of inulin was 10 mg/mL and both were reacted on a rotator at room temperature in the dark for 45 min to allow oxidation of the inulin particles. The reaction was quenched with glycerol (1.8 mL) before centrifuging (RCF = 3270 for 7 min). The oxidized particles were washed three times with PBS pH 7.4 0.01 M (RCF = 3270 for 7 min) and the final oxidized particles were redispersed in PBS to make approximately about 10 mg/mL.

### 2.3. Diamine Coupling of Oxidized MPI Particles

The solution of ethylenediamine (36.84 mg/mL, 100× excess) was prepared in Milli-Q water. The phosphate buffer (0.4 M) was prepared using sodium dihydrogen phosphate hydrate (2.76 g) with Milli-Q water. An equal amount of each was combined and the pH was adjusted using concentrated hydrochloric acid to pH 6.5. The oxidized inulin particles were then redispersed in this buffer (10 mg/mL) and sodium cyanoborohydride solution (180 µL, 5M in 1 M NaOH) was then added to this dispersion and this was further reacted for 18 h. The amine-modified inulin particles are then washed three times using PBS by centrifugation (RCF 3270 for 7 min) and the final modified particles were redispersed in PBS. The variables such as the concentration of ethylenediamine (50–200× excess), molar (0.01–0.2 M) of the buffer, pH (6.5–7.4), duration of reaction (12–36 h), the concentration of sodium cyanoborohydride solution (0.5–1.5×) were considered for optimization.

### 2.4. PCA Loading onto Amine Modified MPI Particles

The amine-modified MPI particles (150 mg) were dispersed in sodium phosphate buffer (pH 7, 0.1 M) and to this was added pyrazine carboxylic acid (0.25 mmol, 93.3 mg). Then EDC (0.312 mmol, 187.3 mg) and NHS (0.025 mmol, 8.9 mg) were added and the whole mixture was reacted for three hours at room temperature. The final modified particles were washed three times by centrifuging (rcf 3270 for 7 min) using PBS and lastly, the final particles were stored in PBS buffer. The variables such as the amount of PCA (1–4×), NHS (1–4×), EDC (1–4×), the way of addition of each ingredients, pH (6.3–10.5), duration of reaction (3–24 h), and the concentration of MPI for the reaction (5–20 mg/mL) were considered for the optimization.

### 2.5. Characterization of MPI-ED-PCA

#### 2.5.1. Fourier Transform Infrared Spectroscopy (FTIR)

FTIR spectra were obtained using Shimadzu IRPrestige-21 FTIR 8400 spectrophotometer (Kyoto, Japan). FTIR was used to characterize the presence of functional chemical groups in the materials. The synthesized MPIEDPCA, MPI was freeze-dried and mixed with KBr powder to form a pellet then the FTIR spectra were obtained in the range of 4000–400 cm^−1^ using 64 scans.

#### 2.5.2. Proton Nuclear Magnetic Resonance (^1^H NMR) Spectroscopy NMR

Proton nuclear magnetic resonance (^1^H NMR) spectroscopy (Bruker Avance III 500 NMR, Bruker, Wissembourg, France) was used to validate the synthesis method and demonstrate that PCA was coupled to modified MPI particles. The ^1^H NMR spectra were recorded using a Bruker Avance II 300, spectrometer operating at 300 MHz. The ^1^H NMR spectra allow us to show that the drug is attached to the inulin backbone and also a means of quantification of the PCA molecules linked to MPI by comparing the integrals of the peak at δ 8.79–9.206, ascribable to the protons of the PCA, to those at δ 3.55–4.25 belonging to the protons of MPI backbone.

#### 2.5.3. Zeta Potential Measurements

Z-potential measurements (mV) were performed at 25 °C using a Malvern Zetasizer Nano ZS instrument (Malvern, Worcestershire, UK), fitted with a 532 nm laser at a fixed scattering angle of 173°. Aqueous solutions of MPIEDPCA (0.1 mg/mL), were analyzed after filtration through a 5-μm cellulose membrane filter. The zeta potential (mV) was calculated from the electrophoretic mobility using the Smoluchowski relationship and assuming that K·a ≫ 1 (where K and a are the Debyes–Hückel parameter and particle radius, respectively).

#### 2.5.4. Thermogravimetric Analysis (TGA)

About (6 ± 0.1 mg) each of MPI and the conjugate were weighed and the TGA analysis was carried out using a Thermogravimetric Analyzer Discovery TGA 550 (New Castle, DE, USA) under a nitrogen flow of 10 mL min^−1^ with a heating rate of 10 °C/min between room temperature and 600 °C.

#### 2.5.5. Differential Scanning Calorimetry (DSC)

DSC of the MPI, PCA and conjugate sample was carried out using a Discovery DSC TA Instrument (model Discovery DSC 2920 (New Castle, DE, USA) calibrated with an indium standard. Dry samples about (2 ± 0.1 mg) were analyzed by heating from room temperature to 250 °C at a rate of 10 °C/min under a nitrogen atmosphere (10 mL min^−1^).

#### 2.5.6. Drug Loading

About 200 μL of the MPIEDPCA conjugates were added to 800 uL of HCl acid (pH 1.2) and heating up the sample at 60 °C for about 5 min in order to break the bond (hydrolysis). After 24 h the sample was centrifuged for 5 min at 4500 rcf. Then the total amount of PCA in the supernatant was evaluated using HPLC after dilution of the test sample to appropriate concentration. The amount of PCA in the sample was analyzed using HPLC system (Shimadzu Corporation, Kyoto, Japan) consisting of a series of LC-20ADXR pumps, SIL-20ACXR autosampler, CTO-20AC column oven and SPD20A variable UV detector set at 268 nm, and C18 Altima 250 mm × 4.6 mm column (5 µm) The mobile phase was a mixture of KH_2_PO_4_ buffer (pH 2.10): Acetonitrile (90:10 *v*/*v*) eluted at a flow rate of 0.9 mL/min.

#### 2.5.7. In-Vitro Cleavage

Artificial Lysosomal Fluid (ALF) and Simulated Body Fluid (SBF) were prepared as reported in the literature [40]. 30 mg MPIEDPCA particles which are equivalent to 3 mL was centrifuged and the supernatant was discarded. Then 1 mL of the release media was used to reconstitute this solid particle and transferred into a dialysis bag. The dialysis was then immersed into 15 mL of release medium in a clear glass bottle containing ALF at (PH 4.5, 5.2 and 6.0) respectively and this was incubated at 37 °C under continuous stirring (50 rpm) in a Benchtop Incubator Shaker. An aliquot of the external medium (1 mL) was collected from the outside of the dialysis bag at different time points and replaced with an equal amount of fresh medium and sink condition was also maintained. The amount of PCA cleave from the MPIEDEDPCA was evaluated by HPLC. Then the % cumulative release from the MPIEDPCA at different pH was determined using HPLC after appropriate dilution of the sample.

#### 2.5.8. Efficient Uptake of FITC Labelled MPI Particles by RAW 264.7 Macrophage Cells

The amine-modified MPI was labelled with 6-[fluorescein-5(6) carboxamido] hexanoic acid (FITC) by using similar PCA coupling protocol but with the PCA exchanged for a molar quantity of the probe. This coupling results in the formation of FITC modified inulin particles (MPIEDFITC). Then RAW 264.7 cells of 1 × 10^5^ cells/well were plated into each 12-well plate at 37 °C, 5% CO_2_, high glucose media and left overnight to allow for proper attachment. After this, the cells were then exposed to FITC labelled MPI (MPIEDFITC) and FITC alone at a concentration of 20 µg/mL for 0.5, 2 and 4 h time point. At each time point, the cells were washed 3× in sterile PBS and then scraped off and spun down at 1000 rpm for 5 min. The cell pellet obtained after this procedure was then resuspended in 100 µL of PBS. To ensure an even and uniform preparation of cells before staining, the cells were spun down at 1000rpm for 5 min in a Cytospin4 cytocentrifuge (Thermoshadon, Cheshire, UK). Slides were then left to dry for 24 h before fixation for 10 min in neutral buffered formalin. Counterstaining and coverslipping were then performed using 4, 6 diamidino-2-phenylindole (DAPI) containing fluorescent mounting media. Images of each time point and treatment were then taken using a Zeiss Elyra PS.1 Laser Scanning Confocal Microscope with Zeiss ZEN lite software (CLSM).

#### 2.5.9. Flow Cytometry Measurements (FACS)

For the flow cytometry analyses, RAW 264.7 macrophage cells (~1 × 10^6^/mL) were seeded into 12 well plates and cultured in high glucose media for 24 h in 37 °C, 5% CO_2._ Then FITC (20 µg/mL) and MPIEDFITC conjugate at 20 µg/mL equivalent of FITC was added to the cells and incubated at 37 °C for 30 min, 2 h and 4 h. After each time point, cells were rinsed with PBS three times scrapped off and spun down (centrifuged) at 1000 rpm for 5 min. The supernatant was discarded and the pellet at the bottom resuspended with 200 µL PBS. Finally, the percent of cells containing FTIC in the suspended cells was analyzed using CytoFLEX^TM^ flow cytometry (Beckman Coulter, Brea, CA, USA). The results were analysed with FlowJo^TM^ software.

## 3. Results/Discussion

### 3.1. Synthesis and Physicochemical Characterization of MPIEDPCA

The use of periodate activation is a common approach used in the covalent attachment of amines to polysaccharides such as inulin, dextran, starch, and cellulose [41,42]. The hydroxyl groups on inulin particles were oxidized using sodium periodate and this produced highly reactive hemiacetal aldehyde that subsequently reacts with an ethylenediamine linker to form a Schiff-base. The labile Schiff-base was reduced to a secondary amine linkage by the addition of sodium cyanoborohydride solution to stabilize the linkage. The first modification results in the introduction of amine pendant group to the inulin particles. The second step involves the coupling of PCA to the amine modified MPI particles via an amide bond. This used EDC/NHS as a coupling agent to activate the carboxylic acid on PCA to react with modified amine forming MPIEDPCA conjugate (Figure 2).

### 3.2. Optimization of Diamine MPI and PCA Coupling

To increase the drug loading onto MPI particles, the syntheses were optimised. Variables including the concentration of ethylenediamine (18.42–73.68 mg/mL, 50–200× excess), molarity (0.01–0.2 M) of the buffer, pH (6.5–7.4), duration of reaction (12–36 h) and the concentration of sodium cyanoborohydride solution (0.5–1.5×) were varied for the ethylenediamine addition. The use of pH 6.5 gives better ethylenediamine (ED) loading than pH 7.4.

**Figure 3:**
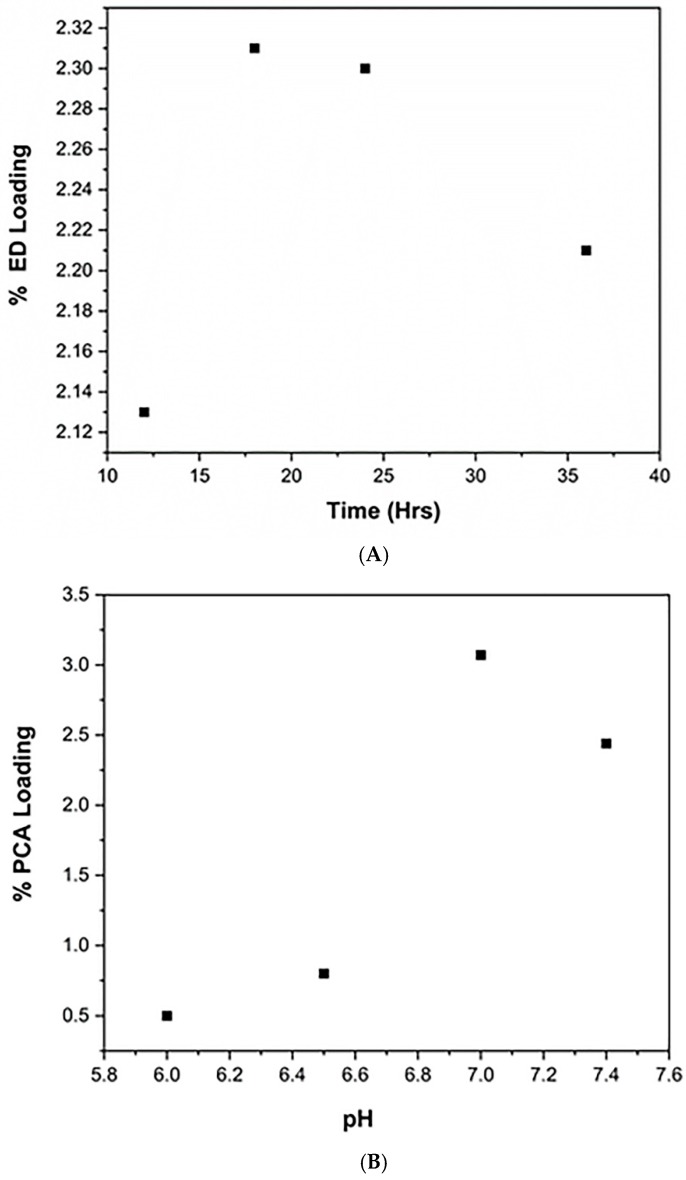
(**A**) Showing the effect of reaction time on ED loading. (**B**) Showing effect of reaction pH on PCA loading.

The optimization isolated the following reaction conditions as best for the ED Coupling; (i) 18 h reaction time; (ii) pH 6.5 and; (iii) 180 μL of sodium cyanoborohydride reagent for 180 mg MPI (Figure 3A).

The PCA coupling was also optimized by varying the amount of PCA (1–4×), NHS (1–4×), EDC (1–4×), the method of addition of each ingredients, the pH (6.3–10.5) of the reaction, the duration of the reaction (3–24 h), and the concentration of MPI for the reaction (5–20 mg/mL). The key results in this optimization showed that overnight and longer reaction times did not result in significant increase in PCA loading and 3 h seem to give a better result. Doubling of PCA concentration, NHS, EDC (X4, X2) did not result in increased loading of PCA. The use of sodium phosphate buffer pH of 7.0 was slightly better than pH 7.4, 6.5 and 6.0 for coupling PCA via the EDC/NHS coupling chemistry (Figure 3B). Consequently, the following reaction parameters were considered optimal for the PCA coupling reaction; (i) 3 h reaction time; (ii) pH of 7.0 for the sodium phosphate buffer used in the coupling reaction and; (iii) the ratio of EDC and NHS remain same as the standard protocol.

### 3.3. Characterization Using ^1^h NMR Spectroscopy

**Figure 4:**
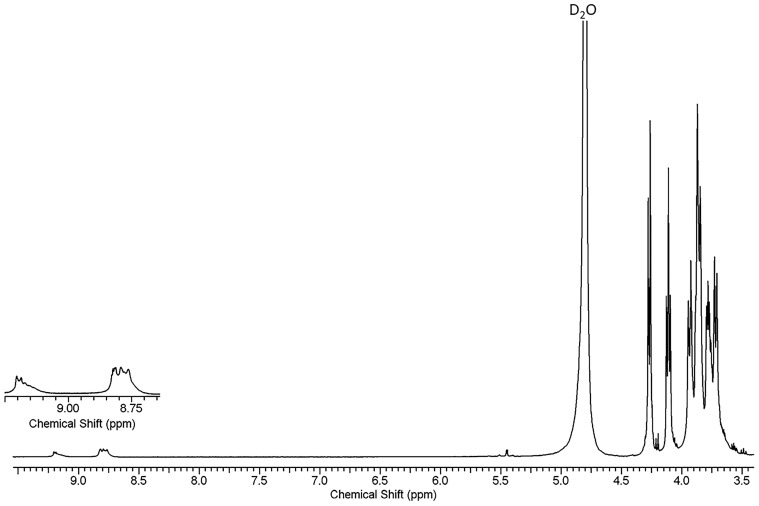
^1^H-NMR spectra of MPIEDPCA confirming PCA attachment to MPI.

The successful formation of the new conjugate was confirmed using^1^H NMR spectroscopy and FTIR. The ^1^H-NMR spectrum of MPIEDPCA is shown in Figure 4 (see Supplementary Materials Figure S2A–C for ^1^H-NMR spectra of MPI, MPIED, and PCA are shown in) and the peaks arising at 8.79–9.2 ppm indicated the successful conjugation of PCA to the amine modified MPI. From the spectrum of MPIED, the degree of derivatization can be calculated by comparing the integral of the peak attributable to ethylenediamine δ 2.74–3.15 (4HEDA, –NH–CH_2_–CH_2_–NH_2_) to the peaks between 3.55 and 4.25 attributed to the MPI protons. Based on this, the amine pendant loading was 3.82% m/m relative to MPI (Supplementary Materials Figures S1 and S2). ^1^H-NMR spectroscopy also allowed quantification of the amount of PCA by comparing the integrals of the peak belonging to the MPI protons δ 3.55–4.25 compared to the proton attributed to the phenyl ring of PCA with δ 8.79–9.2 (Figure 4). The amount of PCA linked to the MPIED was determined using two methods (^1^H NMR and UV spectroscopy). The % loading of PCA calculated using the ^1^H NMR equal to 3.07% m/m of the MPI repeating units and this result is in agreement with HPLC method.

### 3.4. FTIR

**Figure 5:**
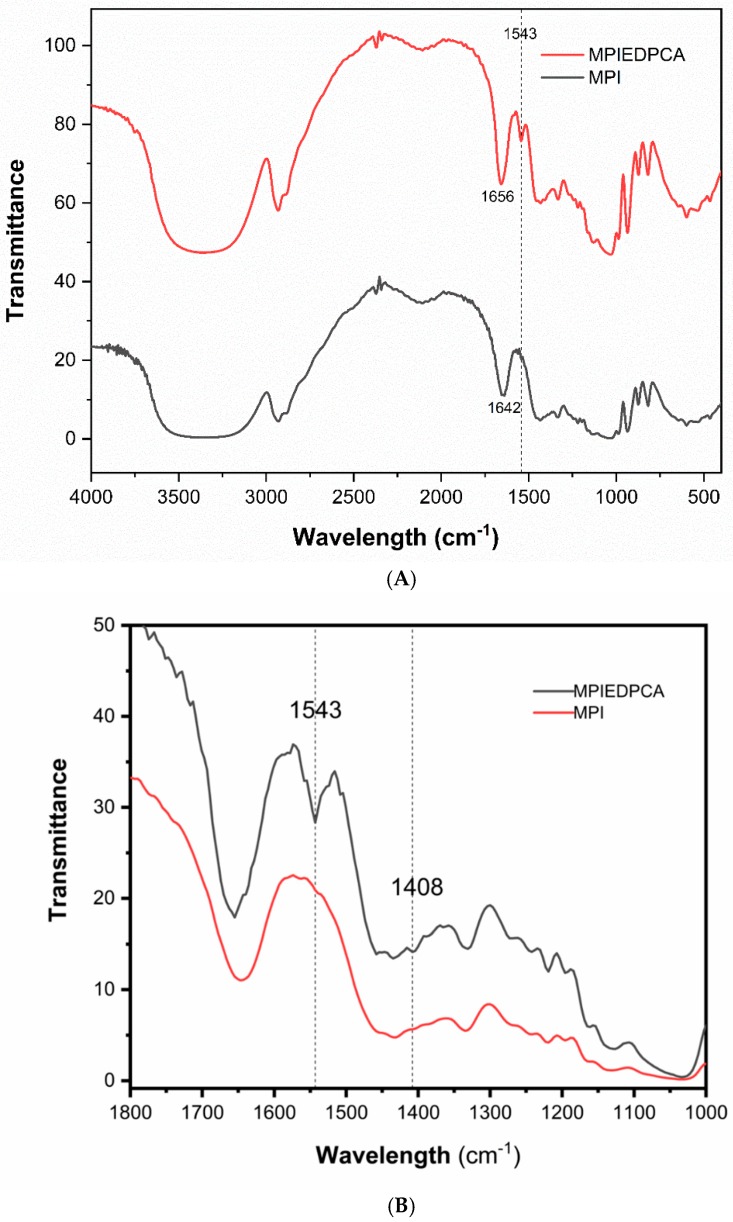
(**A**) The FTIR spectra of both MPIEDPCA and MPI. (**B**) The FTIR spectra of both MPIEDPCA and MPI between 1800 and 1000 cm^−1^.

The attachment of PCA to the amine modified MPI via amide bond was confirmed by FTIR. (Figure 5A,B). The spectra of PCA is characterized by the presence of the OH group of the carboxylic acid with the broad band at 3130 cm^−1^ and a band at 1732 cm^−1^ for the (C=O) carbonyl group (Figure S3, Supplementary Materials) [43]. MPI shows a band at 3363 cm^−1^, 2926 cm^−1^ and 1028 cm^−1^ that can be attributed to OH stretching, aliphatic *CH_2_* stretching and *COC* bending, respectively [44]. The MPI spectra also has bands at 880 cm^−1^, 935 cm^−1^, 992 cm^−1^, 1037 cm^−1^, 1120 cm^−1^ and 1143 cm^−1^ that are sharpened compared to amorphous inulin due to the semicrystalline nature of the MPI [45]. After modification of the MPI, the new conjugate show additional bands at 1543 cm^−1^, which can be attributed to the amide-II (Figure 5B). This result is in agreement with previous literature reports [46,47]. In addition, the presence of a small change in the MPIEDPCA conjugate spectra compared to MPI can be attributed to pyridyl ring of PCA in the final conjugate product with bands at 1408 cm^−1^ as shown in Figure 5B [48]. The broadband of MPI around 1642 cm^−1^ shifted slightly to 1656 cm^−1^ in the conjugate due to the modification of the MPI.

### 3.5. DSC

**Figure 6:**
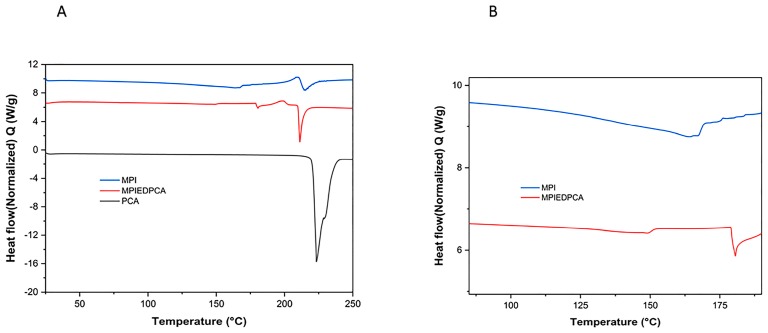
DSC thermograms of MPI, conjugate and PCA (**A**) and MPI and MPIEDPCA (**B**) showing endothermic peak.

PCA shows a typical DSC Profile (Figure 6) characterized by a sharp endothermic peak at 223 °C considered to be the melting point of PCA and in agreement with reported literature [49]. The DSC of the MPI (Figure 6A,B) clearly shows a broad endothermic peak between ~160 °C and 168 °C due to melting of the MPI as expected due to the variety of isoforms determined by different length of inulin chains incorporated into the crystal structure [39] as well as degradation at 214 °C. The DSC of the conjugate varies from MPI alone as there is evidence of a broad endothermic event between 130 and 150 °C that may be a slow glass transition. Also, the main melting transition shifts to 180 °C. This may be due to aromatic interactions between PCA groups on the surface that convert the gradual melting transition of various isoforms and amorphous inulin in the particles into a sharp melting transition (Figure 6B).

### 3.6. TGA

**Figure 7:**
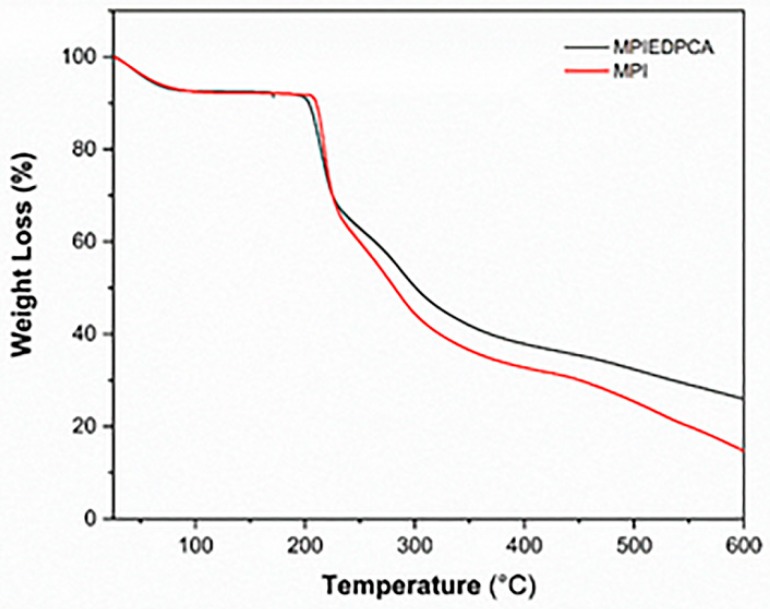
TGA thermograms of MPI and the conjugate MPIEDPCA.

The thermogram of MPI as shown in Figure 7 displays a three-step mass loss as expected. The first event (depression in the TGA curve) occurring from 30 to about 82.5 °C with about 6.85% mass loss can be ascribed to loss of unbound water [50,51]. From 83 °C till about 207 °C the MPI remain stable with no loss in mass. The second major loss (49.5%) started between 207 to 311 °C. This second event can be attributed to the decomposition of the MPI polymer [52]. This TGA result is in agreement with reports from the literature [52]. Evidence of conjugation and covalent linkage was supported by the TGA data. The TGA of the MPIEDPCA shows three steps of mass loss similar to MPI. Initial weight loss for the conjugate was around 6.74%, followed by the second loss of around 43.5%. The decomposition of the conjugate started from 309 °C and the reported weight loss was 22.2%. However, the residual weight (%) at 600 °C for the conjugate and MPI was 25.99 and 14.88% respectively possibly due to the increased carbon-carbon network. Further, the onset of the second mass loss was around 200 °C compared to 207 °C for the MPI. TGA helps to establish that the onset of the main decomposition is shifted down by 7 °C. This can be explained by the fact that oxidation of MPI by periodate can result in reduce molecular weight [53], which can translate to lower degradation temperature couple with the introduction of an amide bond.

### 3.7. SEM

**Figure 8:**
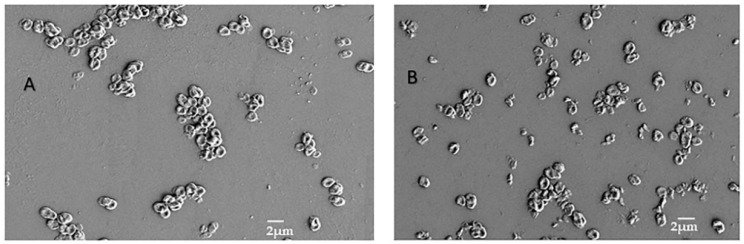
Showing SEM of MPI (**A**) and the MPIEDPCA conjugate (**B**) respectively.

The particle size and morphology of the synthesized MPIEDPCA conjugates were investigated using scanning electron microscopy. The SEM of the MPI microparticles and different conjugates are shown in Figure 8A,B. The SEM images, as previously reported, show that MPI microparticles are of a regular discoid structure comprised of stacks of lamellar sheets with a diameter of about 1–2 μm (spherulite-like discoid shape) [32]. The SEM of the modified MPI conjugates (B) is similar to delta inulin particles both in size and structure. The result of the imaging also gives credibility to the fact that the conjugation reaction didn’t significantly alter the particle size or shape of the conjugate. As the morphology remained constant, the interactions with immune cells are not expected to change significantly. Consequently, the size and surface molecular organization of the synthesized MPI conjugates is expected to contribute to the excellent tropism of these particles towards monocytes and dendritic cells [54].

### 3.8. Zeta Potential

Zeta potential further supports the result obtained by NMR and FTIR. The successful attachment of PCA to the amine modified inulin particles and stability of the conjugate was confirmed using the zeta potential. The measured zeta potential shows remarkable change and shift in the zeta potential of amine-modified inulin particles from 22.8 ± 0.55 mV to 43.8 ± 0.37 mV zeta potential after modification with PCA that further confirms conjugation (Figure S4, Supplementary Materials). Cellular uptake of nano/microparticles is controlled by the size, surface charge, and shape of the nanomaterial [55,56]. Cells typically have a greater preference for positively charged particles compared with negatively charged particles because the cell membrane is assumed to have negative electronic potential [57,58,59,60]. Although this may not be relevant to the case of native MPI, which demonstrates rapid monocyte uptake despite having a negative charge. If anything, this uptake should be even further enhanced by the positive charge imparted by the PCA conjugation.

### 3.9. Drug Loading and pH-Triggered PCA Release

**Figure 9:**
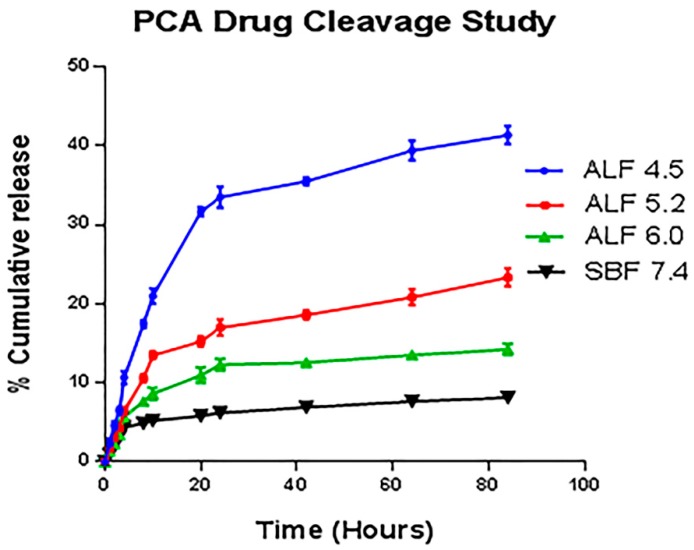
Release of PCA from MPIEDPCA conjugate in ALF media at different pH 4.5, 5.2, 6.0 and SBF 7.4.

To quantitatively assess the PCA content in the MPIEDPCA system. The PCA content was evaluated using reverse-phase high liquid performance chromatography (RP-HPLC) method. After optimization of the amine linker and the PCA attachment, the final MPIEDPCA conjugate was found to have 3.01% of PCA. The HPLC assays further corroborates result obtained using NMR spectroscopy. As shown in Figure 9, the in-vitro release study clearly demonstrates that pH-triggered MPIEDPCA conjugate allows controlled release of the active PCA into the cellular compartments including lysosomes. This system exploits the change in pH between the physiological pH of 7.4 in the bloodstream and the pH value of 4.5 for intracellular lysosomes as a useful strategy for TB drug targeting to immune cells. The in-vitro release profiles clearly shows that MPIEDPCA released the active PCA drug cargo in the intact form for a prolonged period without any burst release. Also, at a pH of 4.5 that mimics the intracellular pH of the lysosomes, over 41% of the drug was released from the conjugate after 6 days. The release was slower with an increase in pH with about 23.3% and 14.2% release for pH 5.2 and 6 respectively after 6 days. Also, the hydrolysis of the conjugate in SBF is slow with a release rate of only 8% over 6 days under conditions modelling blood plasma (pH 7.4) This shows that the MPIEDPCA will have pH tuned release of its drug cargo after entering the monocytes making this system effective drug delivery system for monocytes. This system fulfils the requirement of an effective system for targeting drugs to the infected monocytes such as in TB.

### 3.10. Mechanism of PCA Drug Release

Generally, amides are considered stable to pH-mediated hydrolysis under physiological conditions [61,62] and require enzymes for release. Amides are more stable to acid and base-catalyzed hydrolysis than ester bonds due to the reduced electronegativity of the nitrogen atom in the amide compared to oxygen in the ester. Consequently, the non-bonding electrons on the nitrogen are more likely to be donated to the adjacent carbonyl group, leading to the resonance stabilization and partial double bond character of the bond between the carbonyl carbon and the nitrogen atoms. This reduces the ability of amides to undergo attack by nucleophiles [63]. Specific amides have been found to degrade at mild pH for various electronic and structural reasons. These include monoamides of phthalamic anhydrides [64], cis-aconitamide, maleamide, and citraconamide linkages [65,66], amides that have the nitrogen substituted directly to an aromatic ring [63,67] and Beta-lactams [63]. The attachment of PCA through an ethylenediamine linkage in this research does not contain the key structural features previously identified to undergo pH-mediated degradation under mild conditions. The attachment is similar to a drug delivery system in which methotrexate was coupled to dextran through an ethylenediamine linker and those authors reported release under neutral conditions, though they attributed initially rapid release to incomplete Schiff base reduction and subsequent release was very slow. Due to their use of UV spectroscopy to measure drug release, this may also be attributed to modified methotrexate still attached to the ethylenediamine linker and hydrolyzed dextran oligos [68]. Nonetheless, pH sensitive release is observed for the PCA attached in this system. We have shown that we do have amide attachment and do not have significant levels of ester linkages or supramolecular association under the conditions of the reaction. (Supplementary Materials, Figures S5–S7) As such, there must be something about the connection between the MPI particles and the PCA molecule that supports acid-mediated release.

The linking of pyrazine carboxylic acid through an amide bond to ethylenediamine linked to MPI particles might change the amide stability and susceptibility to hydrolysis for several reasons. Firstly, the pyrazinamide group itself contributes to the release of its acid under acid conditions. This group lacks a proton in the alpha position to the amide carbon, which stabilizes other amides through hyperconjugation. Therefore, in comparison, the pyrazinamide group has increased electropositivity of the carbonyl carbon, assisting the nucleophilic addition of water. The pyrazine ring of the pyrazinamide group also provides electronic and both intramolecular and water hydrogen bonding effects that increase the acidity of the amide N–H bond [69,70,71]. The increased electron donating capacity of the amide hydrogen makes the nitrogen more electronegative and the carbonyl carbon more electropositive, removing some of the stabilizing double bond characters of the carbon-nitrogen bond. This makes both the carbon more susceptible to nucleophilic attack from water and protonation of the nitrogen easier; both steps are key to the progression of the hydrolysis in amide bonds [64]. Nonetheless, the first process in the acid-catalyzed the hydrolysis of amide bonds is the protonation of the carbonyl oxygen atom. The secondary amine of the ethylenediamine linkage will be protonated under neutral and mildly acidic conditions and is conveniently positioned to protonate the carbonyl group. Once protonated, water attack at the carbonyl carbon and nitrogen protonation can occur, the ortho nitrogen of the pyrazine group can then hydrogen bond to the newly formed hydroxyl groups stabilizing the intermediate and promoting amine release and carboxylic acid formation [71].

### 3.11. Kinetic Release Study of PCA

The release data was fitted into the various kinetic release models such as zero order, first order, Hixson–Crowell, Higuchi and Korsmeyer peppers (Table S1, Supplementary Materials) The correlation coefficient of the straight line from the release data analyzed shows that the PCA release profile follows first-order kinetics with a correlation coefficient of 0.9933. Overall the result helped establish that PCA release is controlled by the conjugation of the PCA to the amine modified MPI which results in sustained drug release. This result is consistent with first order process reported by Dang et al. [68] where amide conjugated methotrexate to dextran also exhibited a first order process.

### 3.12. Efficient Cellular Uptake of MPIEDFITC by RAW 264.7 Macrophage Cells

**Figure 10:**
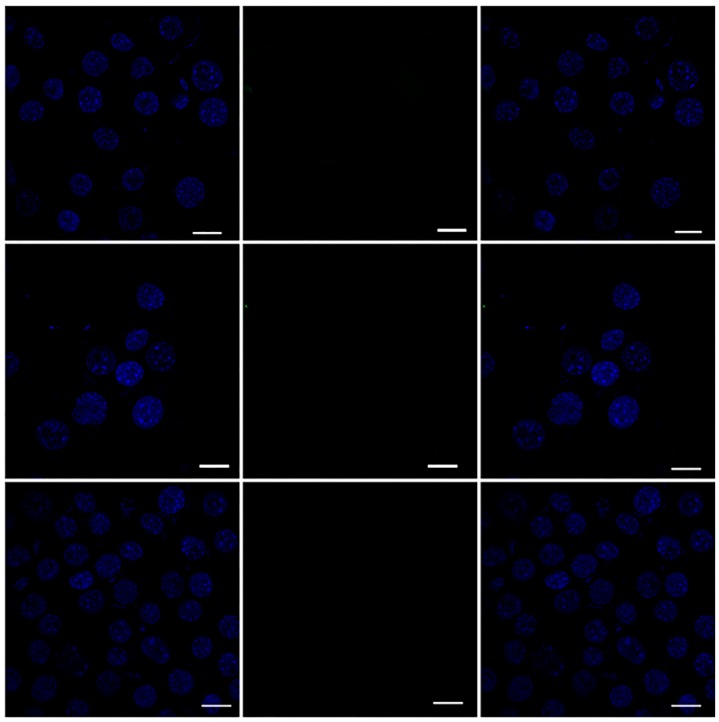
CLSM image of RAW 264.7 macrophage cells incubated with FITC alone. For each panel, the images from left to right show cell nuclei stained by DAPI (blue), the fluorescence of the FITC (Green) and overlays of the two images. Scale bar = 10 μm.

**Figure 11:**
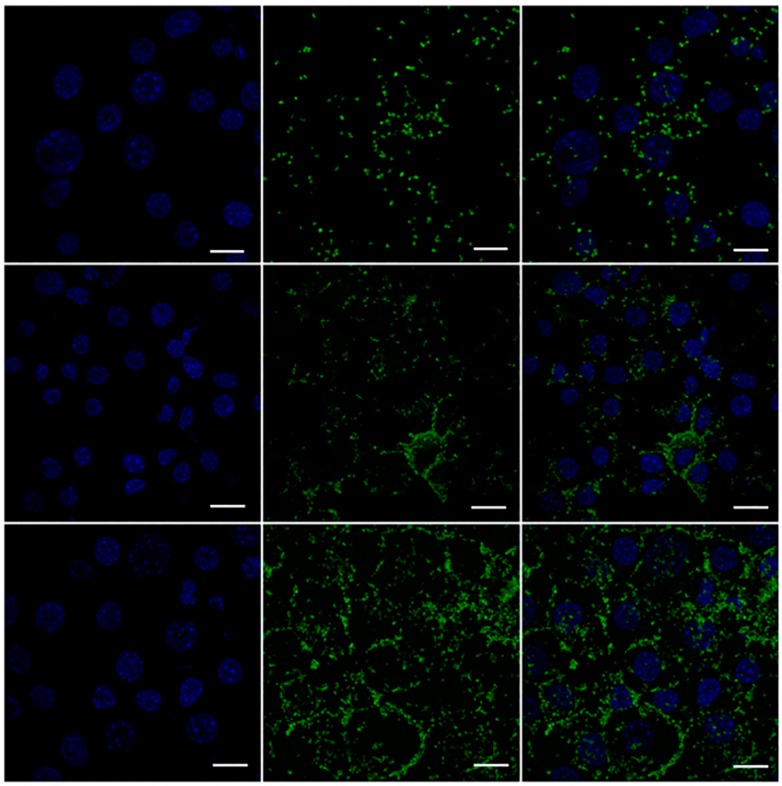
CLSM image of RAW 264.7 macrophage cells incubated with MPIEDFITC. For each panel, the images from left to right show cell nuclei stained by DAPI (blue), the fluorescence of the labelled conjugate (green) and overlays of the two images. Scale bar = 10 μm.

To test whether MPI can serve as a vehicle and delivery tool in primary immune cells, the amine-modified MPI was labelled with FITC in order to assess the cellular uptake of the MPI. Freshly isolated RAW 264.7 cells were cultured in the presence of fluorescent-labelled MPI (containing 20 g/mL of FITC equivalent) or FITC only at various time points). The nuclei of the RAW cells were then stained with DAPI. CLSM was exploited to investigate the efficient targeting power and uptake behavior of the MPI in a time-dependent study. Figure 10 shows that soluble FITC without MPI conjugation did not show any significant uptake into the RAW cells. By contrast, CLSM of the amine modified MPI conjugated with FITC (MPIEDFITC) demonstrated that amine modified MPI promoted the internalisation of the FITC dye (Figure 11). MPIEDFITC was efficiently taken up by the RAW cells and after 30 min incubation the fluorescence was predominantly seen in the cytoplasm. This indicates that the MPI was taken up by the raw cells via endocytosis [72,73] supporting our previous findings [72]. In addition, with increased time of incubation, there was both a significant increase in FITC fluorescence at 2 and 4 h in the cytoplasm and around the nucleus (Figure 11). Appearance of FITC in the nucleus would only be expected to occur once either the individual FITC-labelled inulin polymers themselves become soluble and are able to diffuse into the nucleus or the FITC label is cleaved off the inulin by the acid and/or enzymatic environment of the lysosomes. Consequently, this finding confirms that MPI conjugates are efficiently endocytosed into monocytic cells resulting in intracellular drug release.

### 3.13. Quantification of RAW 264.7 Macrophage Cellular Uptake

**Figure 12:**
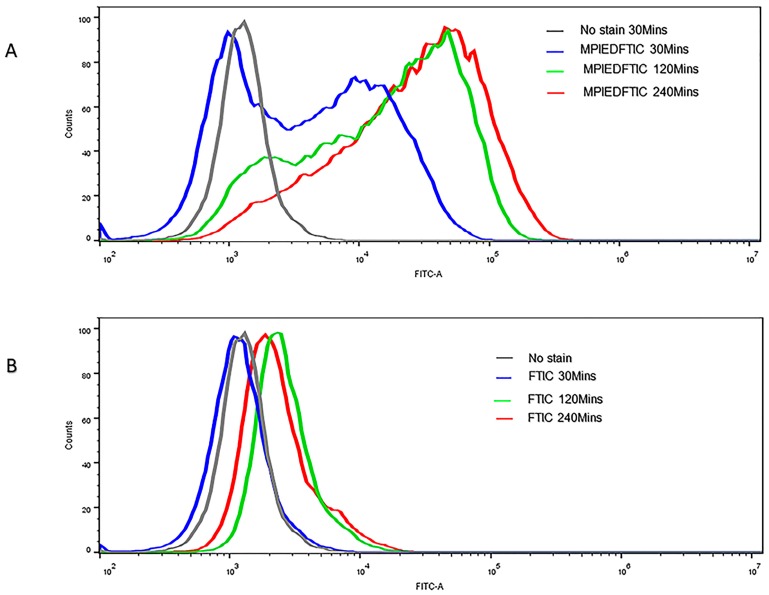
Show flow cytometry analysis (histogram) of RAW 264.7 macrophage cells incubated with FITC-labelled MPI (**A**) and FITC alone (**B**) at a different time point (30 min, 120 min, and 240 min). The histogram of cell count versus FITC intensity (FITC-A denotes FITC area). Data is representative of three sets of experiments.

**Figure 13:**
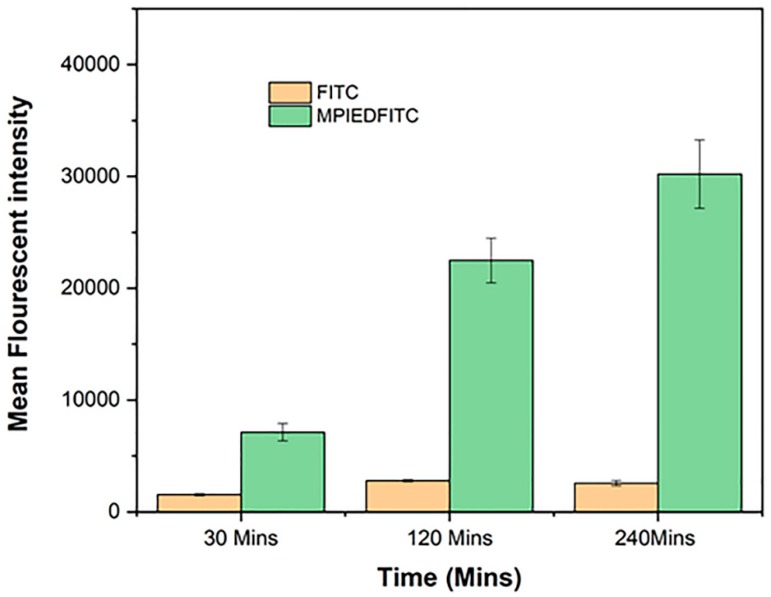
Flow cytometry histogram of cell count versus FITC intensity (FITC-A denotes FITC area). Data is representative of three sets of experiments.

The cellular uptake of MPIEDFITC and FITC was also quantitatively determined by flow cytometry. As shown in Figure 12A,B, histograms of macrophage cells incubated with FITC labelled MPIED conjugate and FITC only at 30, 120 and 240 min. As expected for a microparticle system, the green fluorescence intensity of MPIEDFITC was greatly enhanced when the incubation time of the macrophage cells with particles was increased with a proportional increase in the mean fluorescent intensity of raw cells treated with MPIEDFITC. This increase means that after 4 h incubation, the relative geometrical mean fluorescence intensities of macrophage cells incubated with MPIEDFITC are approximately 10-fold greater than with FITC only stained macrophage cells (Figure 13). The fluorescence of FITC alone is likely due to diffusion across the cell membrane. These results show that FITC and conjugate labelled FITC must have different cellular uptake mechanisms, in agreement with the confocal microscopy. As such, the flow cytometry results confirm the capacity of MPI conjugates to be efficiently internalized by monocytes, a process that can be exploited for the release of drugs to combat *Mtb* that is harboured within phagocytic cells.

## 4. Conclusions

In this study, PCA was effectively coupled to MPI using an ethylenediamine linkage. The amide linking chemistry was stable to extracellular conditions and was readily cleaved once endocytosed by RAW 264.7 monocytic cells. This suggests the conjugate could have potential for intracellular delivery of this antibiotic for the treatment of TB. In particular, the use of MPIEDPCA to target drugs to phagocytic immune cells provides some promise it might be able to be used to overcome multi-drug resistant TB where TB drugs are otherwise actively transported out of the cell by the *Mtb* pathogen.

## Supplementary Materials

The following are available online at https://www.mdpi.com/1999-4923/11/5/243/s1. Figure S1. ^1^HNMR spectra of raw MPI without any drug attachment; Figure S2. (A–C) ^1^HNMR spectra *of* PCA, amine modified MPI and MPIEDPCA; Figure S3. FTIR spectra of PCA; Figure S4. Change in zeta potential due to modification of MPI; Figure S5. ^1^HNMR spectra of reaction product of unmodified MPI and PCA activated with EDC/NHS; Figure S6. ^1^HNMR spectra of reaction product of unmodified MPI and PCA without EDC/NHS; Figure S7. ^1^HNMR spectra of reaction product of amine modified MPI with PCA without EDC/NHS (MPIED + PCA); Figure S8. HPLC chromatogram of PCA; Figure S9. Calibration curve for the pure PCA standard; Figure S10. UV spectrum of both PCA (10 μg) and MPIEDPCA (equivalent amount of PCA); Table S1. Kinetic release models for PCA.
