# Supplementary material for: Design and Characterization of Inulin Conjugate for Improved Intracellular and Targeted Delivery of Pyrazinoic Acid to Monocytes
Source: Pharmaceutics. 2019 May 22;11(5):243. doi: 10.3390/pharmaceutics11050243 (PMC6572292; doi:10.3390/pharmaceutics11050243)
Supplement: Supplementary file 1 [file pharmaceutics-11-00243-s001.pdf]

Supplementary Materials

Design and Characterization of Inulin Conjugate for Improved Intracellular and Targeted Delivery of Pyrazinoic Acid to Monocytes

Franklin Afinjuomo, Thomas G. Barclay, Ankit Parikh, Yunmei Song, Rosa Chung, Lixin Wang, Liang Liu, John D. Hayball, Nikolai Petrovsky and Sanjay Garg

Supplementary Materials: *Supplementary Data for PCA paper*

NMR spectra of the starting MPI and conjugate

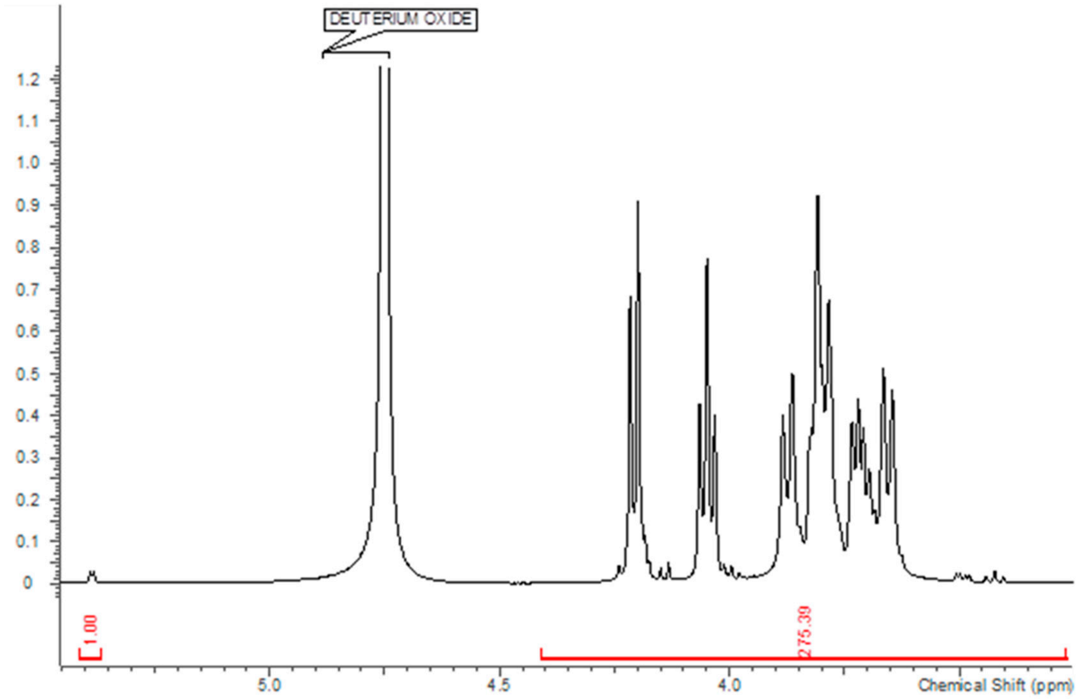

Figure S1. Raw MPI without any drug attachment.

A

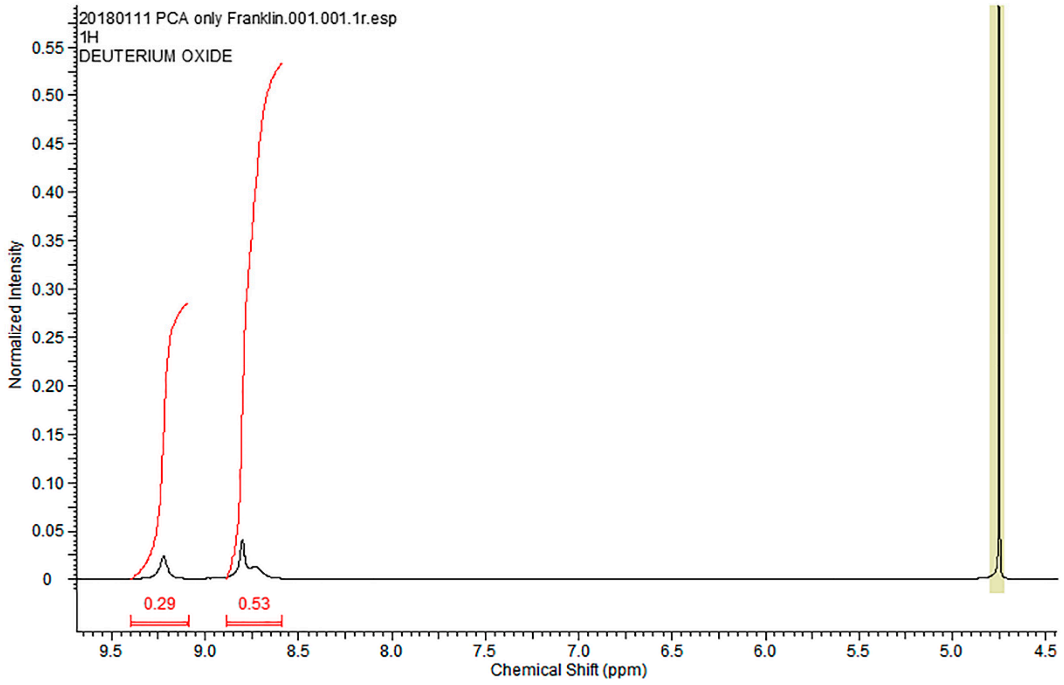

20180111 PCA only Franklin.001.001.1r.esp
1H
DEUTERIUM OXIDE
Normalized Intensity
0.29
0.53
Chemical Shift (ppm)

B

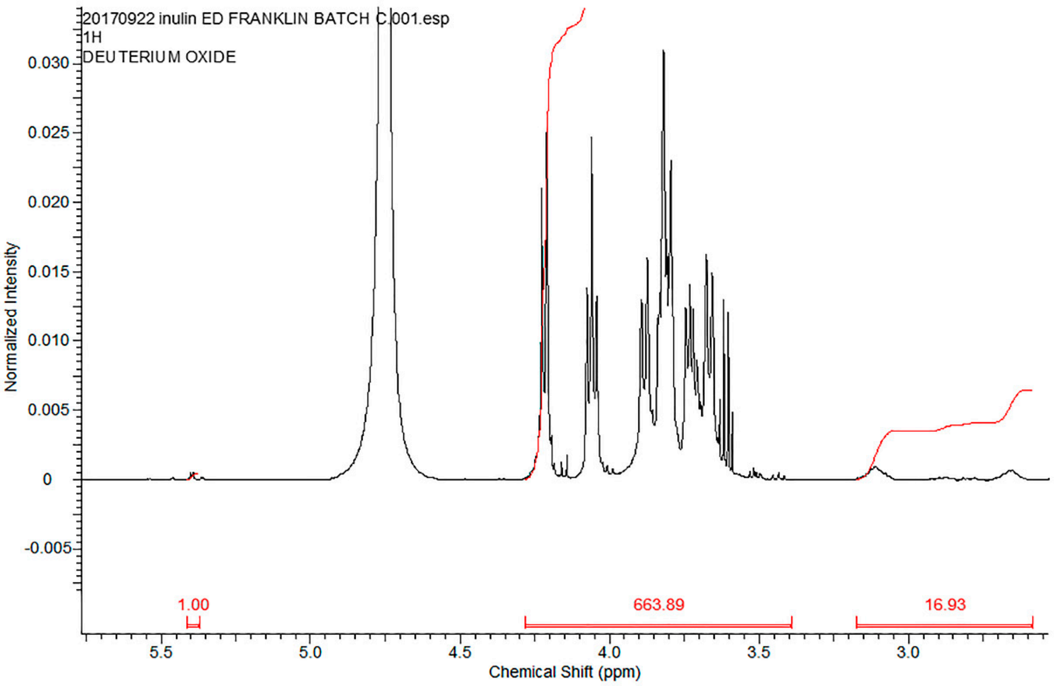

20170922 inulin ED FRANKLIN BATCH C 001.esp
1H
DEUTERIUM OXIDE
Normalized Intensity
1.00
663.89
16.93
Chemical Shift (ppm)

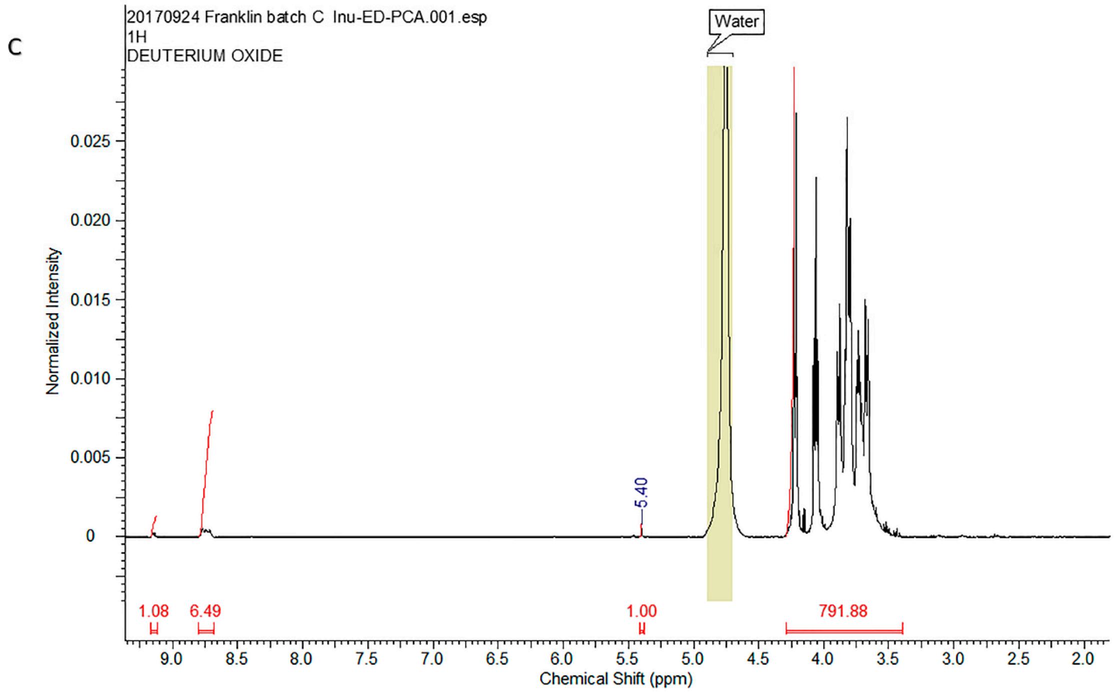

Figure S2. (**A–C**) showing *NMR spectra of* PCA, amine modified MPI and the conjugate.

FTIR

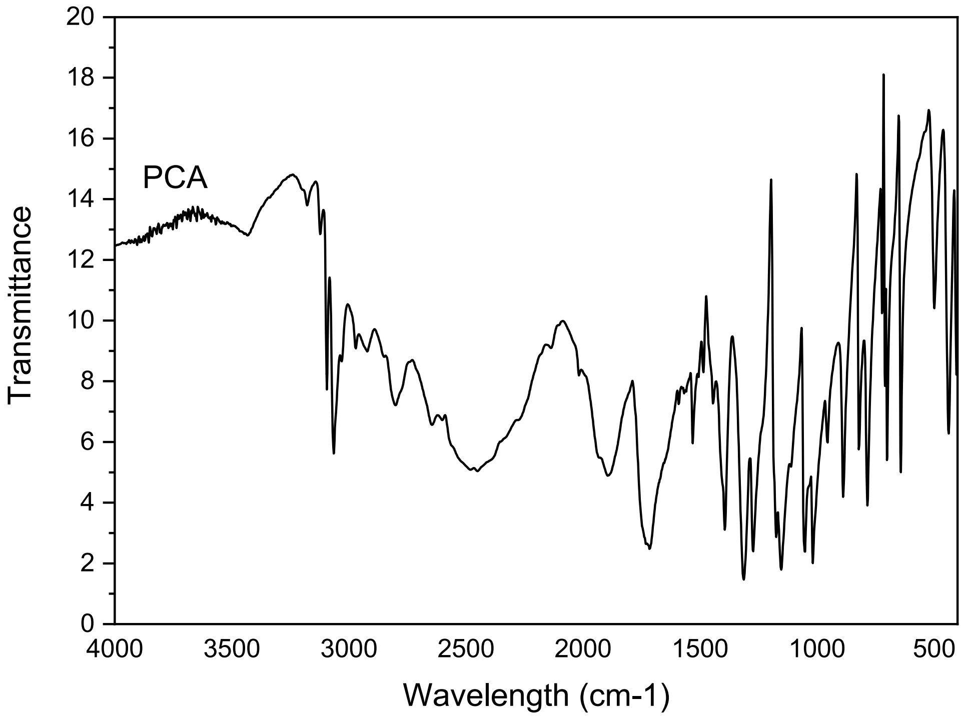

Figure S3 showing the FTIR spectra of PCA.

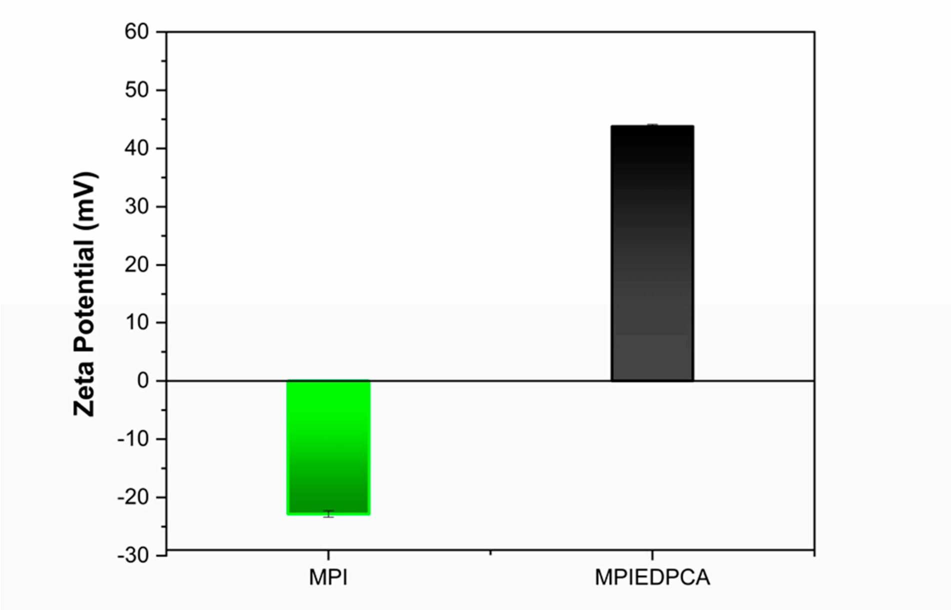

Figure S4 shows change in zeta potential due to modification.

<u>**Experiment on ester and ionic bond**</u>

- No ester and ionic bond formed

To ensure that there is no formation of ester and ionic bond between PCA and inulin 2 further experiments were conducted. First unmodified inulin particles was added to PCA activated with EDC/NHS reaction to see if any PCA attachments will occur with inulin OH (Inulin-PCA) – producing esters. (Figures S5 and S6) Second experiment involves using amine modified inulin with PCA without EDC/NHS (Inulin-ED+ PCA) which can produces ionic ionic bond. The ^{1}H NMR result in both experiment didn't show any PCA attachment

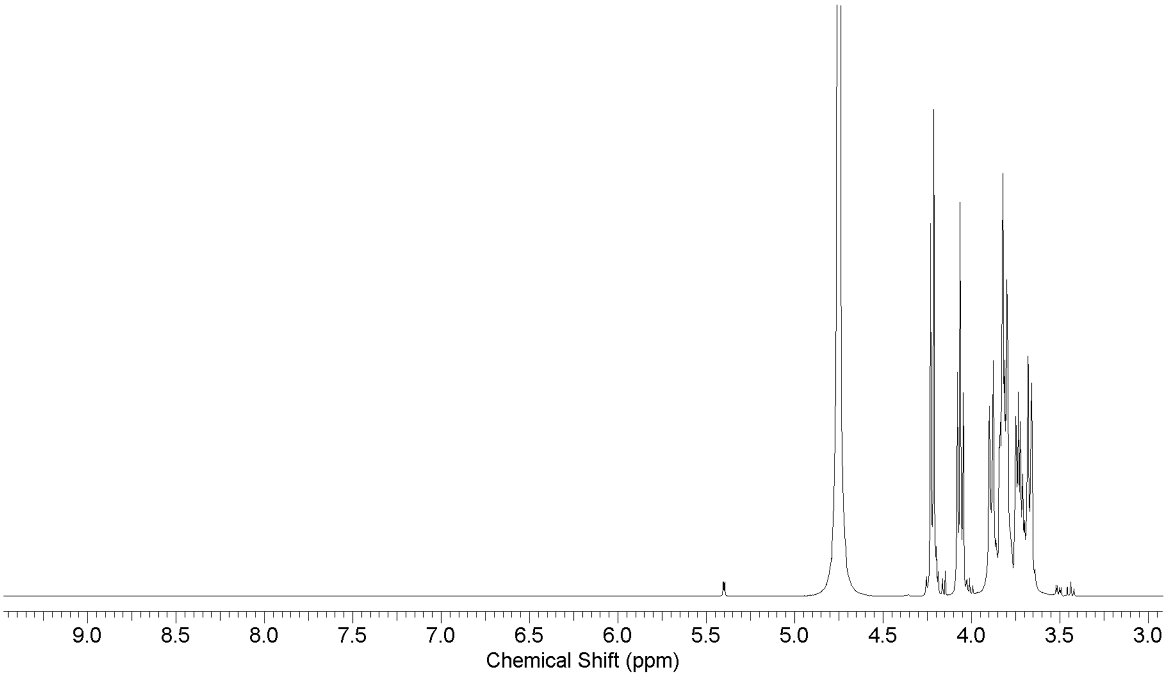

Figure S5 showing the ^{1}H-NMR spectra of reaction product of unmodified inulin particles and PCA activated with EDC/NHS. Figure S3 shows No PCA attachment via ester bond formation.

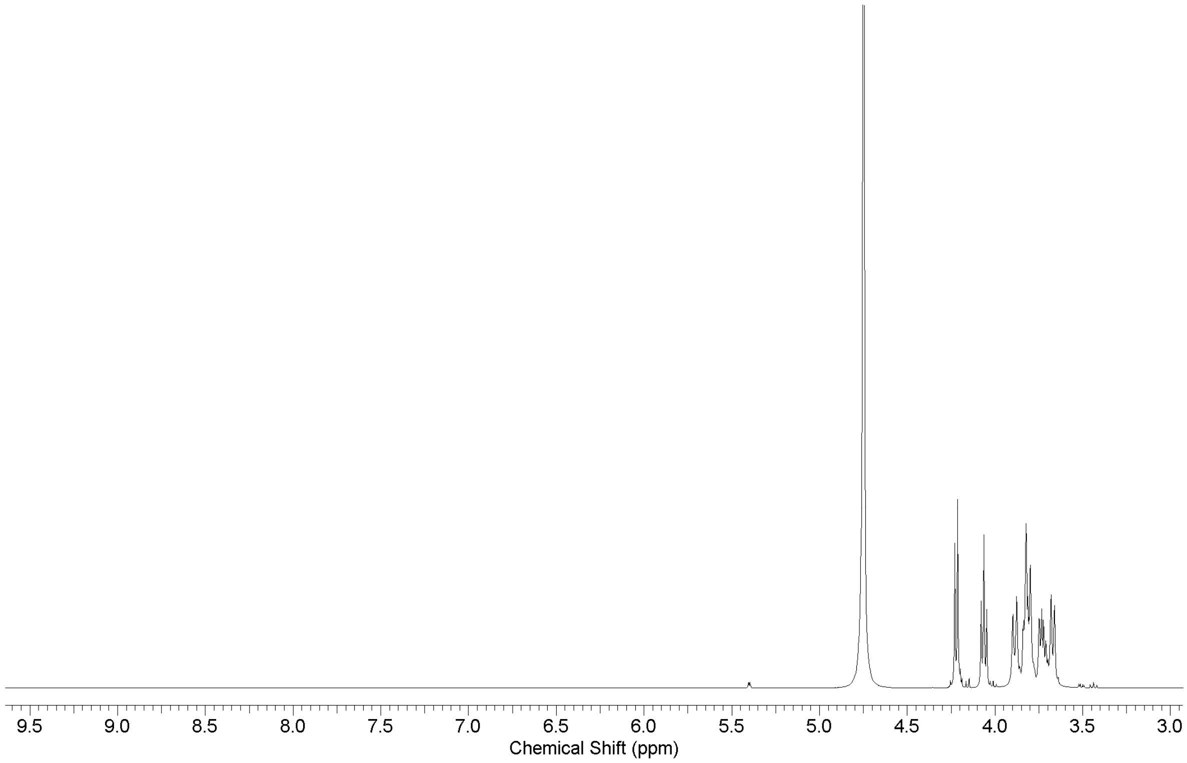

Figure S6 showing the ¹H-NMR spectra of reaction product of unmodified inulin particles and PCA without EDC/NHS. Figure S4 shows No PCA attachment via ester bond formation

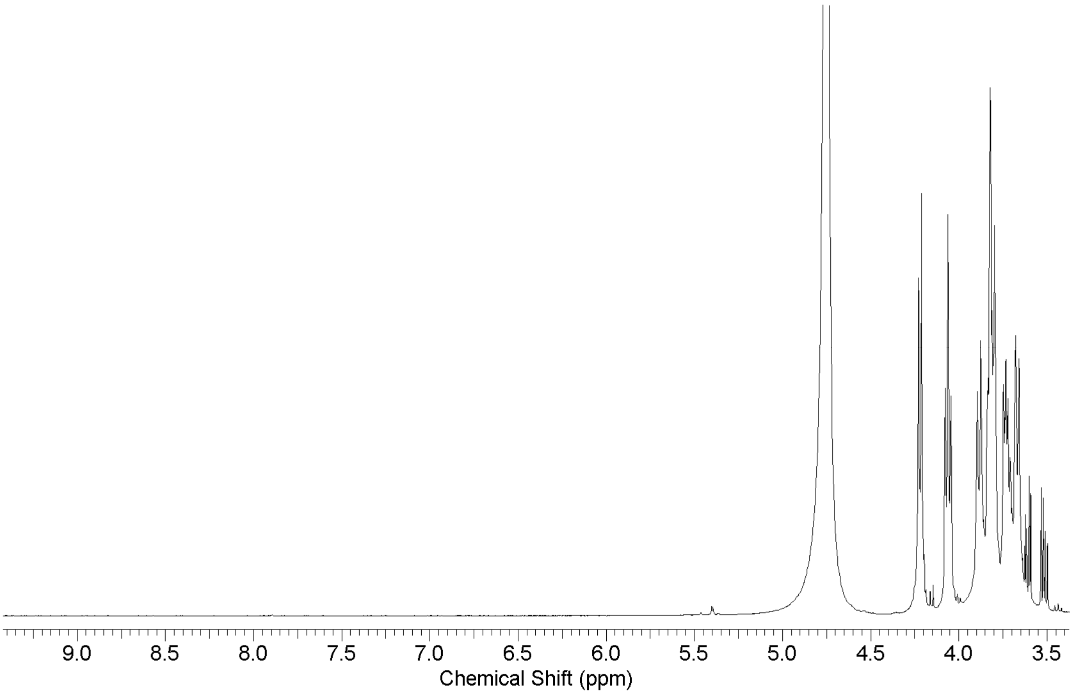

Figure S7 showing the ¹H-NMR spectra of reaction product of amine modified inulin with PCA without EDC/NHS (Inulin-ED+ PCA). Figure S5 shows No PCA attachment via ionic bond.

<u>Preparation of ALF and SBF</u>

The artificial lysosomal fluid was prepared from the reference paper [1].

<u>HPLC Method for the determination of PCA loading via HPLC</u>

1000 μL of the inulin – PCA complexes which is equivalent to 10 mg was added to 1000 μL of HCl acid (pH 1.2) in order to break the bond (hydrolysis) and release the attached PCA. After 24 h the sample was centrifuged for 5 min at 4500 rcf. Then the total amount of PCA in the supernatant was evaluated using HPLC after dilution of the test sample to appropriate concentration. Method adapted from Pitarresi et al [2] The calibration curve was performed by using standard PCA in mobile phase (y = 78953x + 5074 R^2 = 0.9999).

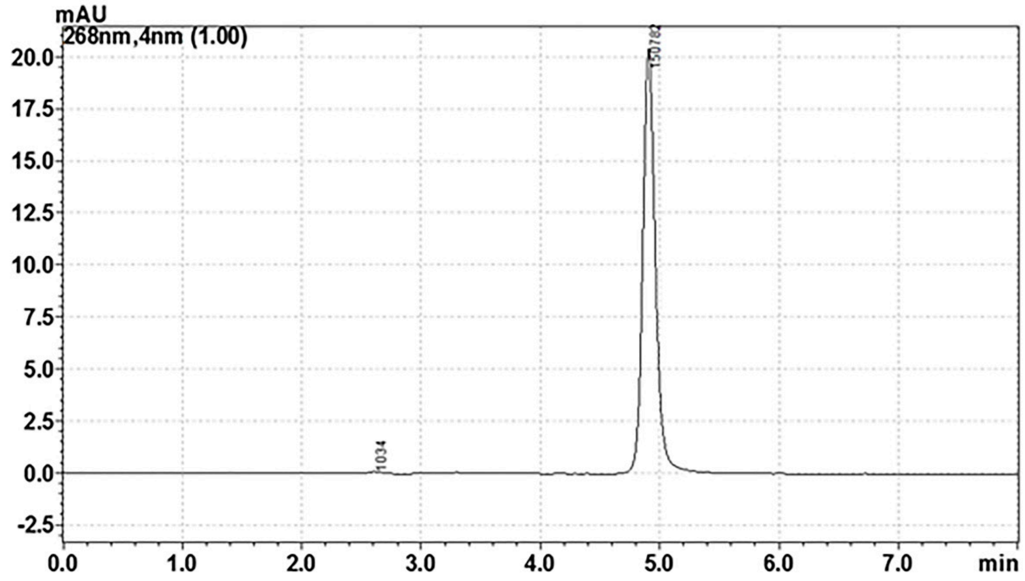

Figure S8. HPLC chromatogram of PCA.

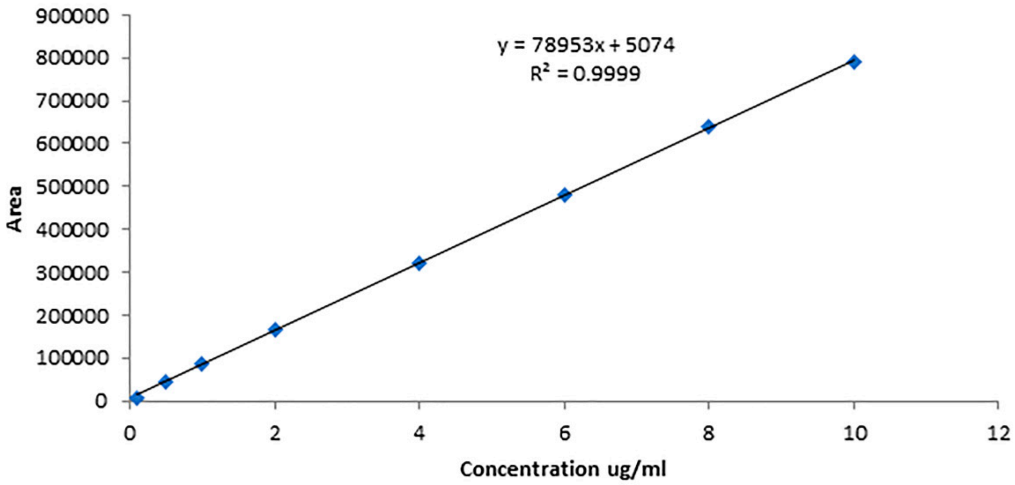

Calibration curve for pure PCA

Figure S9. showing the calibration curve for the pure PCA standard.

CONCENTRATION (µg/mL)	AREA
**0.1**	7771
**0.5**	36339
**1**	74128
**2**	150782
**4**	295842
**6**	446920
**8**	585645
**10**	739117

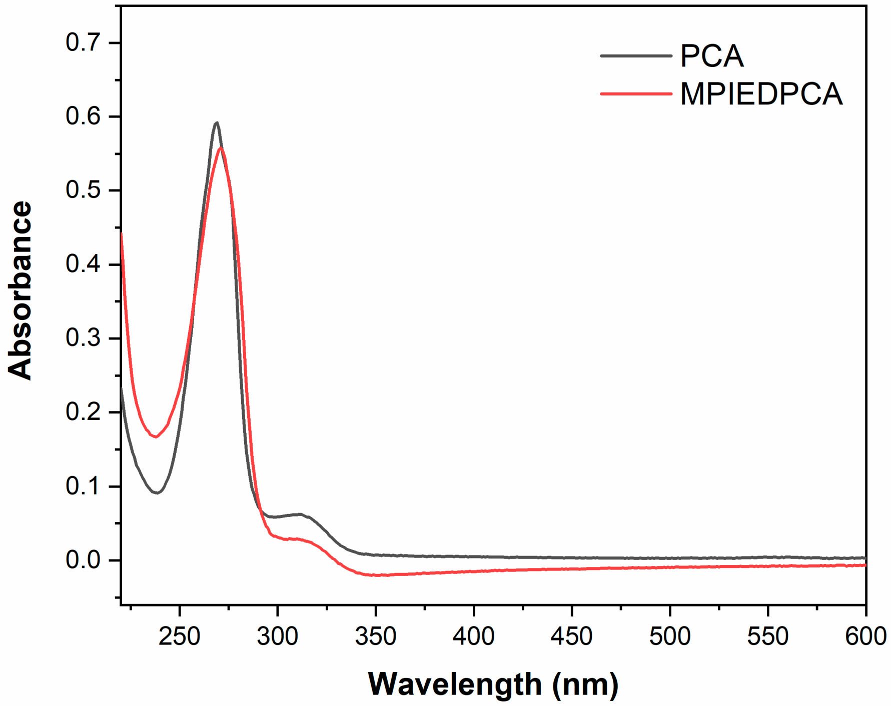

Figure S10. Graph showing that both UV spectrum of both PCA (10 µg) and MPIEDPCA (equivalent amount of PCA). The spectrum is similar when overlaid.

Table S1. kinetic release models for PCA.

Mechanism	R^2
Zero order	0.9557
First order	0.9933
Huguchi	0.9597
Kors peppas	0.9225
Hixson	0.953

References for Supplementary Part

1. Marques, M.; Löbenberg, R.; Almukainzi, M. Simulated Biological Fluids with Possible Application in Dissolution Testing. *Dissolution Technol.* **2011**, 15–28, doi:/10.14227/DT180311P15.
2. Pitarresi, G.; Tripodo, G.; Cavallaro, G.; Palumbo, F.S.; Giammona, G. Inulin-iron complexes: A potential treatment of iron deficiency anaemia. *Eur. J. Pharm. Biopharm.* **2008**, *68*, 267–276.
